# Parent-reported atypical development in the first year of life and age of autism diagnosis

**DOI:** 10.1007/s10803-022-05506-1

**Published:** 2022-04-20

**Authors:** Hannah Waddington, Ella Macaskill, Andrew J. O. Whitehouse, Wesley Billingham, Gail A. Alvares

**Affiliations:** 1 School of Education, Victoria University of Wellington, Wellington, New Zealand; 2 Telethon Kids Institute, University of Western Australia, Perth, Australia; 3 Cooperative Research Centre for Living with Autism (Autism CRC), Long Pocket, Brisbane, Queensland Australia

**Keywords:** Autism spectrum disorder, Diagnosis, Help seeking, Parental concerns, Atypical development

## Abstract

This study examined whether parent-reported atypical development in their child’s first year was associated with age of diagnosis and age when parents first needed to consult a specialist about their child’s development. It involved 423 children who participated in the Australian Autism Biobank. Most parents retrospectively identified ≥ 1 domain of atypical child development. Atypical development in most domains was associated with an earlier age when parents felt specialist consultation was needed. Atypical development in the “gaze abnormalities”, “lack of response to social stimuli”, and “no social communication” subdomains within the social domain was associated with an earlier age of diagnosis, as was atypical development in the “hypo/hypersensitivity” and “preoccupation with parts of objects” subdomains within the stereotyped/restricted behavior domain.

**Supplementary Information:**

The online version contains supplementary material available at 10.1007/s10803-022-05506-1.

Autism spectrum disorder (autism) is a neurodevelopmental condition diagnosed by difficulties with social communication and the presence of restricted and repetitive behaviors, interests and activities (American Psychiatric Association, 2013). Early identification and diagnosis are essential for the provision of early intervention, which is frequently associated with improved developmental outcomes for children with autism and their families (Whitehouse et al., 2019; Zwaigenbaum et al., 2015). There is now good evidence that, for many children, early behavioral signs associated with a later diagnosis of autism can be identified in the first two years of life (Johnson & Myers, 2007; Zwaigenbaum et al., 2015). This has led to increasing use of routine developmental surveillance and autism-specific screening during toddlerhood (Herlihy et al., 2014; Robins et al., 2014; Wetherby et al., 2008; Zwaigenbaum et al., 2015). Despite these efforts, children are generally diagnosed after the age of four (Daniels & Mandell, 2014; van’t Hof et al., 2020).

A greater understanding of the earliest signs of autism has significant potential to support early identification and diagnosis. Parents are often the first to notice and report concerns^1^ regarding their children who will later receive a diagnosis of autism (Chawarska et al., 2007; Guinchat et al., 2012; Sacrey et al., 2015). Several studies have found that parents of children diagnosed with autism retrospectively or prospectively report more early concerns related to autism characteristics than parents of children who do not have autism. This includes concerns about early social communication difficulties and delays (Chawarska et al., 2007; Sacrey et al., 2015; Werner et al., 2005), as well as restricted and repetitive behaviors (Richler et al., 2007; Sacrey et al. 2015; Werner et al., 2005). Some parental concerns that predict an autism diagnosis do not relate specifically to characteristics of autism. These include concerns regarding the presence of challenging behaviors (Sacrey et al., 2015), sleep problems (Sacrey et al., 2015), motor concerns (Chawarska et al., 2007; Sacrey et al., 2015), medical concerns (Richards et al., 2016), and language difficulties/delays (Sacrey et al., 2015; Watson et al., 2007; Werner et al., 2005). Indeed, communication and language difficulties/delays are often the most commonly reported early parental concern in this population (Herlihy et al., 2015; Hess & Landa, 2012; Richards et al., 2016; Turygin et al., 2014).

Several studies have found that parents of children with autism report a greater number of concerns around child behavior and development in the first few years of life than parents of children without autism (Hess & Landa, 2012; Ozonoff et al., 2009; Sacrey et al., 2015; Werner et al., 2005; Zuckerman et al., 2015). Richards et al. (2016), however, found that the total number of concerns did not differ between parents of children with autism and parents of children with other developmental delays. Parents in a prospective study by Talbott et al. (2015) also reported more early concerns if their child had an older sibling with autism but within this “elevated likelihood” group, there was no difference between the number of reported concerns for children who were subsequently diagnosed with autism and those who were not.

Across studies, parents generally first became concerned about their child’s development when they were between 1 and 2 years of age (Chawarska et al., 2007; Coonrod & Stone, 2004; Guinchat et al., 2012). Werner et al. (2005), however, reported that parents of children with autism had significantly more concerns related to social symptoms at 3–6 months than parents of typically developing children, but the number of concerns did not differ from the parents of children with developmental delay until 13–15 months. Ozonoff et al. (2009) and Sacrey et al. (2015) both reported that that the number of concerns after the age of 12 months were more predictive of an autism diagnosis than those reported at 6 months, however, Sacrey et al. (2015) did find that concerns specifically related to sensory and motor skills at 6 months of age indeed predicted an autism diagnosis.

Parental early concerns may also be related to the age at which a child is diagnosed. Concerns about social communication and language have been found to be predictive of an earlier age of diagnosis (Becerra-Culqui et al., 2018; Guinchat et al., 2012; Sicherman et al., 2021; Zablotsky et al., 2017), while concerns about restricted and repetitive behaviors predicted an earlier age of diagnosis in some instances (Sicherman et al., 2021, Zablotsky et al., 2017) and not in others (Becerra-Culqui et al., 2018, Guinchat et al., 2012). In one example, Sicherman et al. (2021) found that parent-reported “severe” presentation in the areas of lack of response to name, lack of gestures, and delayed language was associated with a decrease in age of diagnosis of 33 months compared to both mild presentation or no parental concern in these areas. Few studies have examined other factors that may be related to diagnosis, such as the age at which parents sought professional help (Guinchat et al., 2012; Zablotsky et al., 2017; Zuckerman et al., 2015). Clarifying how other factors relate to diagnosis is important for understanding the diagnostic pathway and, thus, reducing age of diagnosis.

In some prospective studies, parents were regularly interviewed about concerns regarding their child’s behaviour or development (Ozonoff et al., 2009; Sacrey et al., 2015), in others, parents completed a diary regarding their concerns (Talbott et al., 2015). Retrospective studies generally used questionnaires to gather qualitative or quantitative data on the age at which parents first became concerned about their child’s development, and the nature of those concerns (Becerra-Culqui et al., 2018; Guinchat et al., 2009; Zablotsky et al., 2017). Irrespective of these differences in methodology, each of these studies referred to parental “concerns”. Parents may also retrospectively identify early developmental differences in their child, which they did not feel particularly concerned about at the time. An understanding of these earliest parent-reported areas of atypical development could aid in reducing the age of diagnosis.

This current study is the first to examine whether parent-identified atypical child development within the first year of life is related to the timing of parental help seeking and child diagnosis. The study aimed to understand the nature and variety of parent-reported atypical development when their children with autism were aged ≤ 6 months and 7–12 months. It also aimed to examine whether the number of parent-reported domains of atypical development and the type of atypical development were related to the child’s age when parents felt specialist consultation was needed and the age at which their child was diagnosed. This should provide valuable information for clinicians about those early characteristics that may facilitate timely diagnosis and those that do not.

## Methods

### Ethical Clearance and Informed Consent

Ethical approval for this study was provided by human research ethics committees at Princess Margaret Hospital for Children (2014029EP), La Trobe University (HEC16–104), Sydney Children’s Hospital Network (14/SCHN/269), Mater Health Services (14/MHS/212), the University of Queensland (2,014,001,079), and the University of Western Australia (RA/4/1/8184). An ethics exemption was also gained from Victoria University of Wellington for the data analysis. All participants provided written informed consent and were aware that their participation was voluntary and confidential.

### Participants

This study involved analyses of data collected from families who participated in the Australian Autism Biobank (AAB) between 2014 and 2018 (Alvares et al., 2018). Children with autism were eligible to participate in the AAB if they were aged between 2 and 17 years and had a DSM-IV (APA, 2000) or DSM-5 (APA, 2013) confirmed diagnosis of autism.

Children were only included in the current study if they were diagnosed with autism and were under the age of 6 years at the time of participation. This age restriction was employed to increase the chance that parents would be able to accurately recall aspects of atypical child development in the first year of life. Children whose parent did not complete the section of the Family History Questions (FHQ) related to atypical development in the first year of life were also excluded.

### Setting

Participants were recruited through four sites/states in Australia: (a) the Telethon Kids Institute, University of Western Australia in Perth, Western Australia; (b) the Olga Tennison Autism Research Centre, La Trobe University in Melbourne, Victoria; (c) the University of New South Wales in Sydney, New South Wales; and (d) the Lady Cilento Children’s Hospital in Brisbane, Queensland.

### Measures

The children with autism and their parents participated in a large battery of assessments as part of the broader AAB, which are described in Alvares et al. (2018). Here we only describe the measures relevant to the current study.

#### Family History Questionnaire (FHQ)

All measures used in the currently study were drawn from the FHQ. This questionnaire was developed specifically for use with the AAB sample (Alvares et al., 2018). The wider FHQ contains 131 items separated into 8 subsections: questionnaire completion, background information, maternal health, paternal health, pregnancy, birth, child development, and medical history. Only the background information, child development, and medical history subsections were relevant for the current study.

The background information section contained questions about the demographic characteristics of the child including sex, age, diagnosis, and age of diagnosis. It also contained questions about the child’s parents including ethnicity, education, whether the mother or father completed the FHQ, and family income. In the current study, parent ethnicity and education is only presented for the parent who completed the FHQ. There was also a record within the biobank database as to whether or not each child had another sibling with a diagnosis of autism, and the age of that sibling.

The section on child development contained two questions regarding the child’s atypical development. Parents were advised to refer to their child’s “baby book” (a universal health record containing immunizations, records of child health nurse visits, and physical measurements, amongst other health information), if available, for help in answering these questions. The first question was: “*Do you recall anything unusual about your child’s development or behavior during their first 6 months?”* (referred to herein as “atypical development at ≤ 6 months”) and the second was: *“Now, think of the period between 6 and 12 months, can you recall anything unusual about your child’s development or behavior during that time?”* (referred to herein as “atypical development at 7–12 months”). These questions invited parents to describe *any* behaviour that was different from what they would typically expect, rather than explicitly asking about concerns or behaviours that they were particularly worried about. Parents could respond yes or no to these two questions. If they responded yes, there were several lines in which they could provide more details about the specific aspects of atypical development that they noticed at each age.

The only question included from the medical history section was: *“How old was your child when you first felt that there was something wrong with your child’s development and you needed to see a specialist?* (referred to herein as “age when parents felt specialist consultation was needed”). Parents responded with the age they felt specialist consultation was needed, in years and months.

### Data Coding

Parents’ free-text responses regarding atypical development at ≤ 6 months and 7–12 months were coded based on the domains and corresponding subdomains outlined by Guinchat et al. (2012). The adapted domains related to: (a) language, (b) social development, (c) stereotyped/restricted behavior, (d) motor development, (e) behavior/temperament, (f) medical issues, (g) abnormal physiological function (i.e. difficulties with sleeping and feeding), and (h) other idiosyncratic development. Upon coding the data, two additional domains were added to accommodate parent-reported atypical development that did not fit within the existing domains. These related to (i) “atypical physical features” and (j) “unspecified autism concerns”. This resulted in 10 domains of atypical development.

Each domain had between 0 and 9 corresponding subdomains. For example, the subdomains for language included: (a) delayed speech/vocalizations, (b) no speech/vocalizations, (c) poor language comprehension, (d) language regression, (e) lack of language imitation, and (f) other atypical language. Once coding began, several additional subdomains were added to those identified by Guinchat et al. (2012). These included, for example, “delayed social communication” (social domain), “sucking/swallowing” (motor domain), and “self-harm” (behavior/temperament domain). Some subdomains from the Guinchat et al. (2012) article such as incontinency and perceptive abnormalities were also removed because they were not relevant to the parent responses in this sample. Table S1 contains a full list of the included domains and subdomains at each age, each with an example of a relevant coded parent response.

Each parent response was coded for all relevant domains and subdomains. A score of 1 in each domain/subdomain indicated that the aspect of atypical development was present in the response, while a score of 0 indicated that it was not. It was possible for a parent to report atypical development in several subdomains within each domain. For example, atypical development related to both language regression and delayed speech would result in a score of 1 for those two subdomains and the language domain.

One author (EM) coded all parent responses at both ≤ 6 months and 7–12 months and a second author (HW) coded 20% of responses at each age. All disagreements were resolved by consensus between the two authors. The percentage of agreement was calculated using the formula (agreements/[disagreements + agreements]) × 100. The domain-level agreement ranged from 85.0 to 100% at ≤ 6 months and 84.3–100% at 7–12 months. The subdomain-level agreement ranged from 90.0 to 100% at ≤ 6 months and 90.2 − 100% at 7–12 months.

### Data Analysis

Analyses were conducted using the *R* (R Core Team, 2019) and SPSS statistical packages. Descriptive statistics were calculated for all child and parent demographic characteristics as well as the domain- and subdomain-level areas of atypical development.

McNemar’s test was used to examine whether there was a significant change in the proportion of parents reporting at least one area of atypical development at ≤ 6 months compared to 7–12 months. As the distribution for the atypical development at ≤ 6 and 7–12 months was skewed towards 0, the Wilcoxon signed rank test was used to examine if there were any significant differences in the total number of parent-reported areas of atypical development at ≤ 6 months compared to 7–12 months. Negative binomial regressions were used to examine the effect of child sex, the presence of an older sibling with autism, parent education, and family income on the number of parent reported areas of atypical development at ≤ 6 and 7–12 months. It was not possible to examine the effect of parent ethnicity or parent sex due to the small number of participants within at least one category for these variables (i.e. fathers, and all ethnicities except White and Asian).

Ordinary least squares regressions were used to examine whether the age when parents felt specialist consultation was needed and child age of diagnosis differed significantly depending on: (a) the total number of domains of atypical development at ≤ 6 months and 7–12 months, and (b) the presence or absence of atypical development in each specific domain at ≤ 6 months and 7–12 months. The Benjamini-Hochberg (BH) adjustment was used to account for the number of domain-level analyses and to determine statistical significance. Secondary analyses were conducted to determine the effect of the presence or absence of subdomain-level atypical development at ≤ 6 months and 7–12 months on age of diagnosis, only for those subdomains whose corresponding domain was statistically significant after adjustment. To limit the number of comparisons, this secondary analysis was not conducted for the age when parents felt specialist consultation was needed. The BH adjustment was again used to account for multiple analyses at the subdomain level. For analytic reliability, specific domains and subdomains were only included in each analysis if ≥ 10 parents reported atypical development in this area.

### Community Involvement

The Autism CRC Access Committee provides oversight of access to the AAB data. This committee consists of at least six people appointed by Autism CRC, and currently includes two autistic advisors. Access to data is only granted by when the committee can identify a clear potential benefit of the proposed research to the autistic and autism communities.

## Results

### Sample Characteristics

Table 1 provides the demographic characteristics for the 423 participating children and parents. Children were predominantly male. Their mean age was 45 months (3 years, 9 months) and their mean age when they received their autism diagnosis was 37 months (3 years, 1 month). The average age when parents felt specialist consultation was needed was 22 months (1 year, 10 months). Mothers were more likely to have completed the FHQ than fathers. The parents who completed the FHQ were most commonly White, had completed university, and earned more than AUD$104,000 per year in household family income.

**Table 1:** Child and parent demographic characteristics (n = 423).

Demographic characteristic	n (%)/mean (SD)	Missing data
Site where assessment occurred		0
Western Australia	154/423 (36.4%)	
Victoria	90/423 (21.3%)	
New South Wales	142/423 (33.6%)	
Queensland	37/423 (8.7%)	
Child sex		4
Male	333/419 (79.5%)	
Female	86/419 (20.5%)	
Older sibling with autism diagnosis	57/421 (13.5%)	2
Child age	45.1 months (SD = 11.8 months)	0
Child age when parents felt specialist consultation was needed	22.6 months (SD = 11.0 months)	17
Child age when diagnosed	37.7 months (SD = 12.1 months)	27
Parent relationship to child		0
Biological mother	402 (95%)	
Biological father	21 (5%)	
Parent ethnicity		24
White	275/399 (68.9%)	
Aboriginal	2/399 (0.5%)	
Asian	73/399 (18.3%)	
Māori or Pacific Islander	5/399 (1.2%)	
Other	44/399 (11.0%)	
Parent education		18
< 12 years	34 (8.4%)	
12 years	55 (13.5%)	
Diploma/Trade	102 (25.1%)	
University	215 (53%)	
Family income		26
≤$60,000	81 (20.4%)	
$60,000 - $104,000	116 (29.2%)	
>$104,000	155 (39.0%)	
Prefer not to say	45 (11.3%)	

Note: Parent education and parent ethnicity relate to the parent who completed the FHQ

### Atypical Development at ≤ 6 Months and 7–12 Months

These analyses were conducted to understand the frequency and type of atypical development reported by parents for their child’s first year of life. McNemar’s test indicates that the proportion of parents reporting at least one area of atypical child development at 7–12 months was significantly greater than at *≤* 6 months (*p* < 0.001). The median number of domains of atypical development at ≤ 6 months was 0 (*range* = 0–5) while the median number of domains of atypical development at 7–12 months was 1 (*range* = 0–6). A Wilcoxon signed rank test indicated that the number of domains of atypical development reported by parents at 7–12 months was significant higher than at ≤ 6 months (*Z* = -5.852, *p* < 0.001).

The number and percentage of parent-reported domains and subdomains of atypical development at ≤ 6 and 7–12 months are reported in Table 2. At ≤ 6 months the most common areas of atypical development were behavior/temperament, abnormal physiological function, and the stereotyped/restricted behavior. At 7–12 months the most common areas of atypical development were the social, motor, and behavior/temperament domains.

**Table 2:** Number and percentage of parents reporting atypical development in each domain- and subdomain when their children were ≤ 6 and 7–12 months (n = 423)

Domain-/Subdomain	≤ 6 months			7–12 months	
	n	%		n	%
Reported at least one area of atypical development across domains	191	45.1%		264	62.4%
**Language**	**23**	**5.5**		**74**	**17.5**
Delayed speech/vocalizations	10	2.4		42	10.0
No speech/vocalizations	11	2.6		20	4.7
Poor language comprehension	0	0		2	0.5
Language regression	2	0.5		10	2.4
Lack of language imitation	0	0		4	0.9
Other	0	0		2	0.5
**Social development**	**50**	**11.8**		**95**	**22.5**
Delayed social communication	14	3.3		6	1.4
No social communication	6	1.4		17	4.0
Gaze abnormalities	28	6.6		32	7.6
Poor social interaction	8	1.9		28	6.6
Lack of response to social stimuli	9	2.1		33	7.8
Social/non-verbal regression	0	0		4	0.9
Lack of social imitation	0	0		3	0.7
Other	6	1.4		15	3.6
**Stereotyped/restricted behavior**	**57**	**13.5**		**87**	**20.6**
Stereotyped movements	15	3.6		26	6.2
Need for routine/rituals	7	1.7		7	1.7
Stereotyped/restricted interests	6	1.4		22	5.2
Preoccupation with parts of objects	0	0		19	4.5
Hypo/hypersensitivity	42	10		53	12.6
**Motor development**	**41**	**9.7**		**93**	**22**
Motor delay	16	3.8		76	18.0
Hypotonia	12	2.8		8	1.9
Hypertonia	2	0.5		5	1.2
Motor regression	0	0		2	0.5
Lack of motor imitation	0	0		1	0.2
Swallowing/Sucking	8	1.9		3	0.7
Other	12	2.8		16	3.8
**Behavior/Temperament**	**85**	**20.1**		**91**	**21.6**
Lack of attention and interest	17	4.0		25	5.9
Hyperactivity	5	1.2		7	1.7
Passivity	19	4.5		19	4.5
Tantrums/opposition	7	1.7		13	3.1
Unsettled/crying/anxiety	41	9.7		22	5.2
Aggression/violence	0	0		5	1.2
Self-harm	1	0.2		3	0.7
Extreme attachment to caregiver	9	2.1		15	3.6
Other	10	2.4		11	2.6
**Medical issues**	**39**	**9.2**		**26**	**6.2**
Disorder	40	9.5		5	1.2
Sickness	4	0.9		12	2.8
Other	16	3.8		10	2.4
**Abnormal physiological function**	**59**	**14**		**47**	**11.1**
Sleeping	43	10.2		31	7.3
Feeding	25	5.9		24	5.7
**Atypical physical features**	**5**	**1.2**		**5**	**1.2**
**Unspecified concerns related to autism**	**2**	**0.5**		**1**	**0.2**
**Other idiosyncratic development**	**15**	**3.5**		**25**	**5.9**

Note: Items in bold are domains while indented, non-bolded items are subdomains. There were no subdomains for atypical physical features and unspecified concerns related to autism

McNemar’s tests indicate that the proportion of parents reporting atypical development related to the language (*p* < 0.001), social (*p* < 0.001), stereotyped/restricted behavior (*p* < 0.001) and motor (*p* < 0.001) domains at 7–12 months was significantly greater than at ≤ 6 months. There was no significant difference in the proportion of parents reporting atypical development at ≤ 6 months and 7–12 months for the behavior/temperament, abnormal physiological functioning, medical, or other idiosyncratic development domains. Analyses were not conducted for the atypical physical features or unspecified characteristics of autism domains as < 10 parents reported atypical development in this area at ≤ 6 months and/or 7–12 months.

### Atypical Development and Demographic Factors

These analyses examined whether demographic factors influenced the number of parent-reported areas of atypical development in the first year of life. Table S2 shows the results of the negative binomial regressions examining the effect of demographic factors on the number of parent-reported areas of atypical development at ≤ 6 or 7–12 months. Child sex, the presence of an older sibling with autism, parent education, and family income were not related to the number of parent-reported areas of atypical development at either age.

### Domain-level Atypical Development and Age when Parents Felt Specialist Consultation was Needed

The following analyses were conducted for the 406 children for whom parents reported an age when parents felt specialist consultation was needed. These analyses examined the influence of: (a) the number of domains of atypical development and (b) the presence of atypical development in each domain on the age at which parents felt they needed to see a specialist regarding their child’s development.

**Fig. 1:**
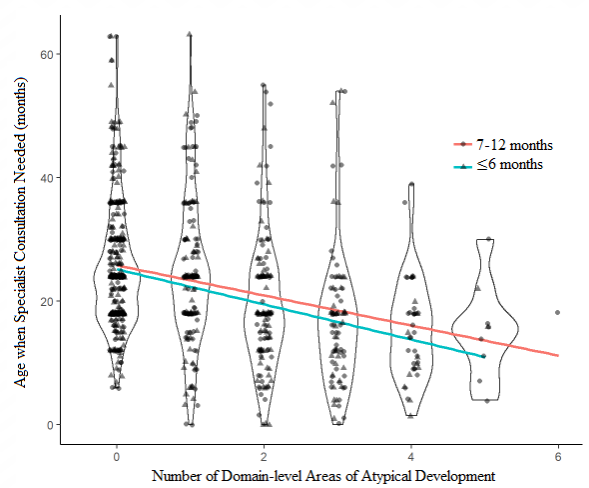
Violin plot of the number of domain-level areas of atypical development and age when specialist consultation was needed

The violin plot in *Fig.* 1 indicates the effect of total number of parent-reported domains of atypical development at ≤ 6 months and 7–12 months on the age when parents felt specialist consultation was needed. The number of domains of atypical development at both ≤ 6 months and 7–12 months were significant predictors of the age when parents felt specialist consultation was needed. At ≤ 6 months, each additional domain of atypical development was associated with a mean decrease in the age when parents felt specialist consultation was needed of 2.85 months (*CI*: -3.70, -1.99, *p* < 0.001). At 7–12 months, each additional domain of atypical development was associated with a mean decrease in the age when parents felt specialist consultation was needed of 2.44 months (*CI*: -3.18, -1.70, *p* < 0.001).

**Fig. 2:**
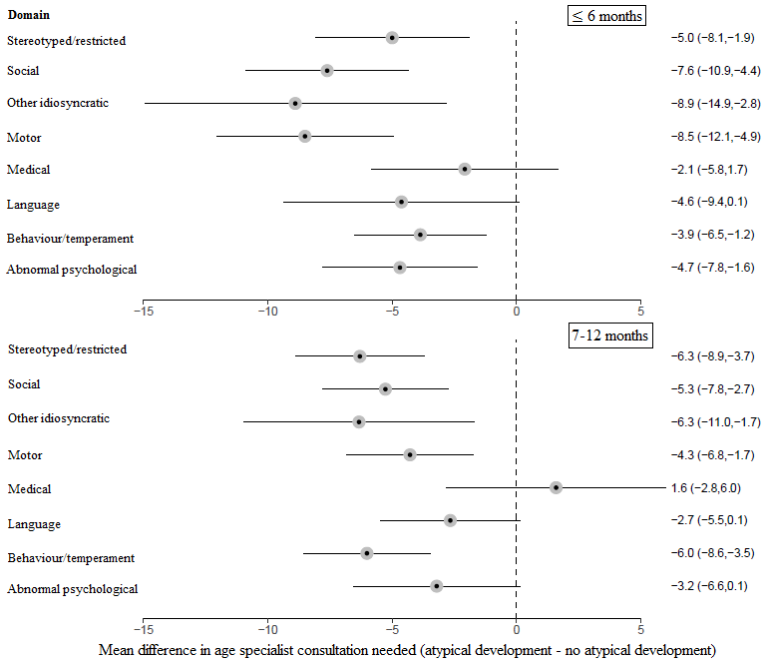
Forest plot of the mean difference in the age specialist consultation was needed depending on whether parents reported, or did not report, each domain level area of atypical development

*Figure* 2 shows the difference in the age when parents felt specialist consultation was needed when parents reported atypical child development in a specific domain at ≤ 6 months and 7–12 months compared to when parents did not report atypical development in that domain. At ≤ 6 months, the mean age when parents felt specialist consultation was needed was significantly earlier in the stereotyped/restricted behavior (*p* = 0.005), social (*p* < 0.001), other idiosyncratic development (p = 0.006), motor (*p* < 0.001), behavior/temperament (*p =* 0.006), and abnormal physiological domain (*p* = 0.006) domains when parents reported atypical development compared to when they did not. There were no significant differences in the parent-reported age when parents felt specialist consultation was needed depending on whether they did, or did not, report atypical development in the language and medical domains.

At 7–12 months, the mean age when parents felt specialist consultation was needed was significantly earlier in the stereotyped/restricted behavior (*p* < 0.001), social (*p* < 0.001), other idiosyncratic development (*p* = 0.010), motor (*p* = 0.003), behavior/temperament (*p* < 0.001), and abnormal physiological domain (*p* = 0.006) domains when parents reported atypical development compared to when they did not. There were no significant differences in the parent-reported age when parents felt specialist consultation was needed depending on whether they did, or did not, report atypical development in the abnormal physiological, language and medical domains.

### Domain-level Atypical Development and Age of Diagnosis

The following analyses were conducted for the 396 children for whom an age of diagnosis was provided. These analyses examined the influence of: (a) the number of domains of atypical development and (b) the presence of atypical development in each domain on the child’s age of diagnosis.

**Fig. 3:**
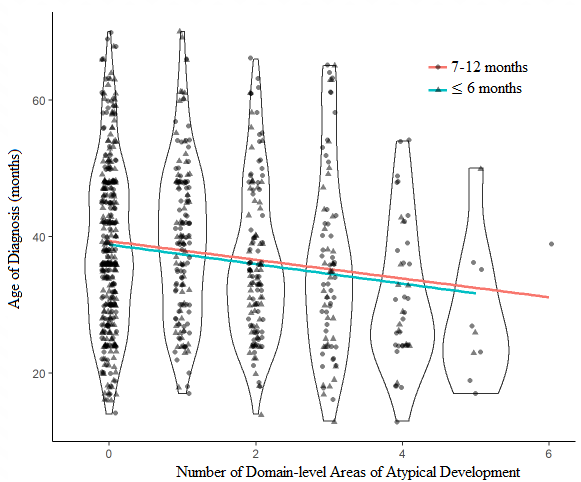
Violin plot of the number of domain-level areas of atypical development and age of diagnosis

The violin plot in *Fig*. 3 indicates the effect of total number of domains of atypical development at ≤ 6 months and 7–12 months on child age of diagnosis. The number of domains of atypical development at both at ≤ 6 months and 7–12 months were significant predictors of age of diagnosis. At ≤ 6 months, each additional domain of atypical development was associated with a mean decrease in age of diagnosis of 1.42 months (*CI*: -2.41, -0.45, *p* = 0.004). At 7–12 months, each additional domain of atypical development was associated with a mean decrease in age of diagnosis of 1.37 months (*CI*: -2.22, -0.52, *p* = 0.002).

**Fig. 4:**
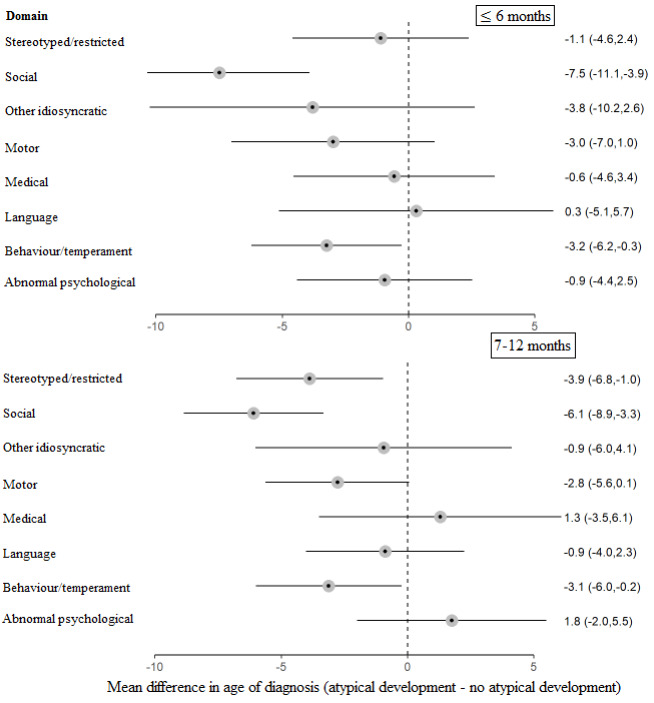
Forest plot of the mean difference in age of diagnosis depending on whether parents reported, or did not report, each domain level area of atypical development

*Figure* 4 shows the difference in age of diagnosis when parents reported atypical child development in a specific domain at ≤ 6 months and 7–12 months compared to when parents did not report atypical development in that domain. After adjustment for multiple comparisons, the association between atypical development in the behavior/temperament domain and age of diagnosis was no longer significant at either ≤ 6 months (*p* = 0.109) and 7–12 months (*p* = 0.109). The only significant difference at ≤ 6 months was in the social domain, where mean age of diagnosis was earlier for children whose parents reported atypical development compared to those whose parents who did not (*p* < 0.001). At 7–12 months, the mean age of diagnosis was significantly earlier in the social (*p* < 0.001) and the stereotyped/restricted behavior (*p* = 0.048) domains for children whose parents reported atypical development compared to those who did not. There were no significant differences for any other domains.

### Subdomain-level Atypical Development and Age of Diagnosis

These analyses examined the influence of subdomain-level parent-reported areas of atypical development on age of diagnosis, for those areas where significant differences were observed at a domain level. Thus, the analyses were conducted for the subdomains within the social domain at ≤ 6 and 7–12 months and the subdomains within the stereotyped/restricted behavior domain at 7–12 months. If atypical development in a particular subdomain was not reported for ≤ 10 participants at each age, then it was not included in the analysis. Thus, in the social domain, gaze abnormalities were included at ≤ 6 and 7–12 months, delayed social communication was included at ≤ 6 months only, and poor social interaction, “other” social behaviors, no social communication, and lack of response to social stimuli were included at 7–12 months only. In the stereotyped/restricted behavior domain, the need for routine/rituals was the only subdomain that was excluded.

**Fig. 5:**
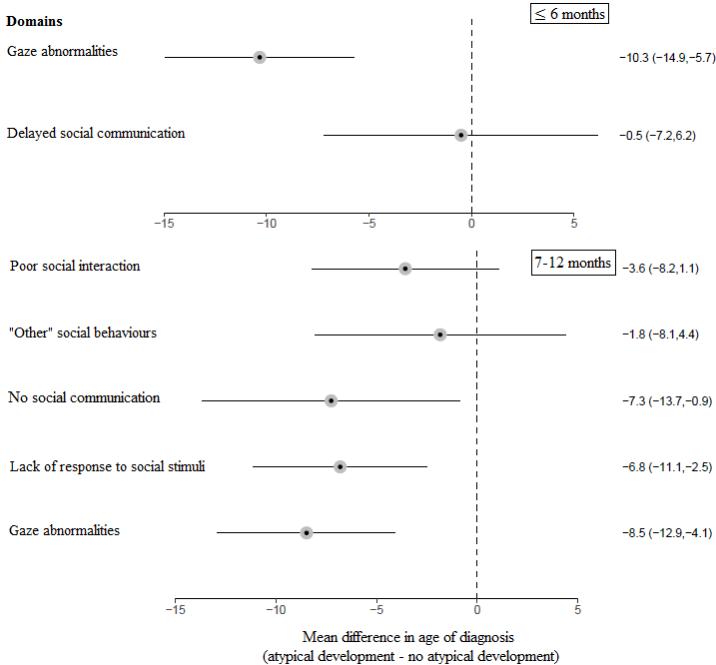
Forest plot of the mean difference in age of diagnosis depending on whether parents reported, or did not report, atypical development in each subdomain within the social domain

*Figure* 5 shows the difference in age of diagnosis when parents reported atypical child development in a specific subdomain within the social domain at ≤ 6 months and 7–12 months compared to when parents did not report atypical development in that subdomain. At ≤ 6 months, children were diagnosed early when their parents reported gaze abnormalities (*p* < 0.001) compared to when they did not. At 7–12 months, the mean age of diagnosis was earlier when parents reported no social communication (*p* = 0.026), lack of response to social stimuli subdomain (*p* = 0.002), and gaze abnormalities (*p* < 0.001), compared to when they did not these aspects of atypical development. None of the other social subdomains was significantly associated with age of diagnosis.

**Fig. 6:**
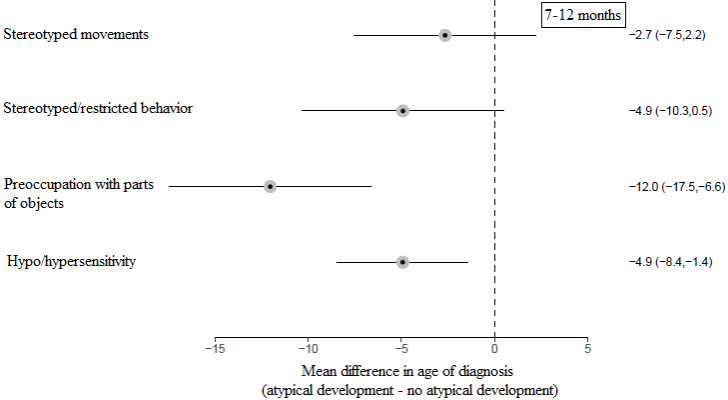
Forest plot of the mean difference in age of diagnosis depending on whether parents reported, or did not report, atypical development in each subdomain within the stereotyped/restricted behavior domain at 7–12 months

*Figure* 6 shows the difference in age of diagnosis when parents reported atypical child development in a specific subdomain within the stereotyped/restricted behavior domain at 7–12 months compared to when parents did not report atypical development in that subdomain. The mean age of diagnosis was earlier when parents reported hypo/hypersensitivity (*p* = 0.006) and preoccupation with parts of objects (*p* < 0.001) compared to when they did not. None of the other stereotyped/restricted behavior subdomains were significantly associated with age of diagnosis.

## Discussion

This was the first study to examine the association between parent-reported atypical child development in the first year of life, the age when parents felt a specialist needed to be consulted, and age of autism diagnosis. For each additional parent-reported domain of atypical development, the average age when parents felt specialist consultation was needed reduced by more than 2 months and the average age when their child was diagnosed reduced by more than 1 month. Parent-reported atypical development in most domains was associated with a significant decrease in the age when parents felt specialist consultation was needed, but only parent-reported atypical development in the social domain at ≤ 6 months and the social and stereotyped/restricted behavior domain at 7–12 months were associated with a significant decrease in the age of diagnosis.

Previous studies have found that parents first identified concerns about their child’s development between 1 and 2 years of age (Charwaska et al., 2007; Coonrod & Stone, 2004; Guinchat et al., 2012). In our study, however, 45% of parents retrospectively identified at least one area of atypical development at ≤ 6 months, which increased to 62% at 7–12 months. On average, parents only felt that they needed to consult a specialist when their child was almost 2 years of age, so it appears that they were not sufficiently concerned by this atypical development to consider further action. The focus on parental concern in previous literature, therefore, may have overlooked these earliest parent-reported signs of atypical development.

Parents were more likely to report at least one domain of atypical development at 7–12 months compared to ≤ 6 months. This may be because children aged 7–12 months are expected to show a wider range of more salient developmentally appropriate behaviors, meaning that parents may be more likely to notice developmental differences (Maenner et al., 2013). It is also possible that many children with autism do not show noticeable signs of atypical development at ≤ 6 months, meaning there could simply be fewer behavioral differences for parents to observe. Thus, more research is needed to understand these earliest developmental differences.

As the number of parent-reported domains of atypical development increased, the age that parents felt specialist consultation was needed and the age their child was diagnosed both considerably decreased. These results match prior research showing that as the number of clinician-identified behavioral features of autism increased, the child’s age of diagnosis decreased (Maenner et al., 2013). Parents who notice fewer areas of atypical development may be more likely adopt a “wait and see” approach than those who notice many areas of atypical development (Caronna et al., 2007). Frontline professionals may also be more likely to refer children whose parents report many aspects of atypical development for further diagnostic assessment. It is also possible that children who display more domains of atypical development show a combination of behaviours that are more easily recognized by professionals as being related to autism, which is associated with earlier diagnosis (Beccera-Culqui et al., 2018; Richards et al., 2016).

Parent-reported atypical development in most areas was associated with an earlier age at which parents felt specialist consultation was needed. This partially contrasts with the results of Guinchat et al. (2012), who reported that some early parental concerns were related to parents seeking professional help earlier, while others were associated with delayed help-seeking. In the present study, the presence of atypical parent-reported language and behavioral development at ≤ 6 and 7–12 months and atypical physiological development at 7–12 months did not prompt parents to seek help earlier. Parents might deem some behavioral, feeding, and sleeping difficulties to be developmentally appropriate for young children, and thus might not consider atypical development in these areas to warrant specialist input (Zablotsky et al., 2017). In contrast, several other studies have found an association between communication delay and timing of help-seeking (Guinchat et al., 2012; Zablotsky et al.,2017) so the relationship between early parental communication concerns and help seeking remains unclear.

The only domains associated with an earlier age of diagnosis were atypical social development at ≤ 6 and 7–12 months and restricted/repetitive behavior at 7–12 months. The social and restrictive/repetitive behavior domains correspond to the core characteristics of autism (American Psychiatric Association, 2013), while the other domains could relate to other co-occurring behaviors or conditions (Soke et al., 2018). For example, atypical development in these other areas could signal other neurodevelopmental conditions, including ADHD (Rao & Landa, 2014). Thus, it appears that only the early aspects of atypical development that most closely corresponded to the autism diagnostic criteria led to an earlier autism diagnosis, regardless of how early parents first sought help or the presence of atypical development in other areas.

Within the social and restricted/repetitive behavior domains, only certain subdomains were associated with an earlier age of diagnosis. These were gaze abnormalities at ≤ 6 and 7–12 months, and a lack of response to social stimuli, no social communication, hypo/hypersensitivity, and preoccupation with parts of objects at 7–12 months. Becerra-Culqui et al. (2018) and Sicherman et al. (2021) similarly found that parental concerns about gaze abnormalities were associated with an earlier age of autism diagnosis. No previous studies appear to have specifically examined preoccupation with parts of objects but, in contrast with the current findings, Sicherman et al. (2021) found that sensory hyperreactivity was associated with a delayed age of diagnosis. These aforementioned behaviors are amongst the most well-known early signs of autism and are frequently included in early screening tools (Frazier et al., 2017; Oosterling et al., 2009). Therefore, these signs might be most easily recognised during first consultations with clinicians and by those performing diagnostic assessments. Further, children who display atypical development in these areas may have a clearer presentation than those with other symptom profiles, meaning that diagnosis may be more straightforward (Whitehouse et al., 2018).

The present findings offer several practical implications. First, it appears that some parents do retrospectively report atypical development related to the core characteristics of autism in their child’s first year of life, even if they did not consider these signs sufficiently concerning to warrant professional input. These parent-reported autism-specific areas of atypical development were also predictive of earlier autism diagnosis. This provides further evidence that practitioners who regularly visit families in the early years should treat parental reports of atypical development seriously, rather than employing a “wait and see” approach (Barbaro et al., 2011; Caronna et al., 2007). Second, only certain aspects of atypical social development and restricted/repetitive behavior were associated with an earlier age of diagnosis. This suggests that clinicians may benefit from greater education regarding the lesser-known signs to help decrease the age of diagnosis for children without behaviors aligning with common conceptions of autism. Third, while over half the parents identified at least one aspect of atypical development at 7–12 months, the average age of diagnosis was just over 3 years. This highlights the significant gap between the age that parents first notice developmental differences and the age that their child is diagnosed (Becerra-Culqui et al., 2018; Maenner et al., 2013). This diagnostic delay can cause significant frustration for parents and prevent timely access to evidence-based early intervention.

This study had several strengths. First, it involved a large sample of children with clinically confirmed diagnoses of autism. Second, the children participating in this study were younger than those in previous similar research (Becerra-Culqui et al., 2018; Guinchat et al., 2012; Sicherman et al., 2021), reducing the likelihood of poor parental recall regarding early atypical child development. Third, the free-text response format allowed parents to report any aspect of atypical development in their own words, rather than forced-choice questionnaires, which can limit or bias parent responses. Finally, the focus on early atypical development – rather than early *concerns* – allowed parents to identify behaviors that occurred at a younger age, before they were considering seeking professional input. These earliest developmental deviations could provide important insights into the first signs of autism in infants, facilitating timely detection and diagnosis.

As there was no comparison group without autism included in this study, future research should determine whether parent-reported early atypical development is predictive of children receiving an autism diagnosis. Future research should also examine which specific aspects of the diagnostic pathway contribute to the reduction in age of diagnosis. The earlier age of diagnosis is unlikely to be solely due to earlier intentions to seek specialist consultation on the part of the parents. This is because parent-reported atypical development in many areas was associated with an earlier age at which they felt specialist consultation was needed, while only those areas of atypical development related to characteristics of autism were associated with an earlier age of diagnosis. It is not clear, however, whether this reduction is due, for example, to the wait time for a diagnostic assessment, to the type or number of professionals seen before a diagnosis was given, or to professionals responding in a proactive way during consultation (Zuckerman et al., 2015). The parents in this study may have been particularly motivated to seek help for their child's atypical development through the medical system. Future research should examine whether these findings are replicated for individuals with more limited trust in the health system. Finally, in future, researchers could examine whether the total number of areas of atypical development *within* each particular domain is predictive of age of diagnosis. For example, whether having a wide variety of language concerns is more predictive of an earlier diagnosis than just one language concern.

The use of retrospective parent-report was a limitation of this study. Although this was mitigated somewhat by only including parents of children under the age of 6 years, it is possible that some parents had difficulty recalling aspects of the timing and nature of their child’s early atypical development. As all children in this study were diagnosed with autism, it is also possible that parents were subject to confirmation bias. That is, parents whose children have a diagnosis of autism may be more likely to recall behaviors associated with autism in the first year of life. Parent did, however, also recall many behaviors unrelated to the characteristics of autism. While we collected data on the age at which parents felt that specialist consultation was needed, it was not clear whether this was also the age at which parents actively sought help. Further, restricting the sample to parents of children under 6 years necessarily excludes those children who were diagnosed after this age, and may have had different patterns of early development. Also, it was not possible to examine the effect of ethnicity or parent sex on the number of parent-reported areas of atypical development due to limited sample sizes in one or more groups. Finally, the demographic characteristics of this sample may not represent the Australian population, as the majority of respondents were female, White, had completed university, and reported higher than average household incomes. Although both parent education and family income were not predictive of the number of parent-reported domains areas of atypical development in this sample, it is possible that another sample of parents with less education or financial resources may have reported different or fewer areas of atypical development and may not have been as easily able to access specialist consultation or diagnostic services.

In the present study, many parents retrospectively reported signs of atypical development in the first year of life for their children who were later diagnosed with autism. The number of parental concerns was related to a decrease in both the age parents felt specialist consultation was needed, and the age their child was diagnosed. Parent reported atypical development related to core symptoms of autism was associated with an earlier age of diagnosis, but atypical development in other areas was not. These results emphasize the importance of seeking, and valuing, parental input regarding their child’s early development.

## Electronic Supplementary Material

Below is the link to the electronic supplementary material.

Supplementary Material 1

## Footnotes

1 The term “concern” is commonly used in the literature. It refers to a behavior/sign that parents have noticed which appears developmentally different from their child’s peers or expected child development (Zablotsky et al., 2017). It does not imply that the behaviours themselves are negative or undesirable. Parents may often, but not always, intend to seek specialist help or consultation in relation to these concerns.
