# Supplementary material for: Parent-reported atypical development in the first year of life and age of autism diagnosis
Source: J Autism Dev Disord. 2022 Apr 20;53(7):2737–48. doi: 10.1007/s10803-022-05506-1 (PMC10290580; doi:10.1007/s10803-022-05506-1)
Supplement: Supplementary file 1 — Supplementary Material 1 [file 10803_2022_5506_MOESM1_ESM.docx]

Table S1.

*Domains and subdomains with illustrative quotes for atypical development at* ≤ 6 months and 7 – 12 months*.*

| Domain/Subdomain | Illustrative quote | | |
| --- | --- | --- | --- |
|  | ≤ 6 months |  | 7 – 12 months |
| **Language** |  |  |  |
| Delayed speech/vocalisations | *“Did not… make happy noises until 12 weeks”* |  | *“Language … was not developing on par with others* |
| No speech/vocalisations | *“Never really babbled”* |  | *“Wasn't talking”* |
| Poor language comprehension | N/A |  | *“Delayed understanding”* |
| Language regression | *“Said 'hello' twice at 5.5 months, never heard from again”* |  | *“Had said 'Mum and Dad' early then stopped everything but 'Mum' at 11 months”* |
| Lack of language imitation | N/A |  | *“Never… copied sounds we were making”* |
| Other | N/A |  | N/A |
| **Social development** |  |  |  |
| Delayed social communication | *“Minimal or no social smile - really needed to do something funny to make laugh”* |  | *“Very limited smiling/giggling”* |
| No social communication | *“Did not smile”* |  | *“He never waves and points his fingers to show something”* |
| Gaze abnormalities | *“After looking back on photos of [child] we noticed he was not looking at us - he was looking behind us”* |  | *“Between 10-12 months reduced eye contact”* |
| Poor social interaction | *“If I got a toy to show him or engage him with, he would cry”* |  | *“Not interested in other children at mother's group”* |
| Lack of response to social stimuli | *“Wouldn't look at mum or turn head to voice”* |  | *“No response when being called”* |
| Social/non-verbal regression | N/A |  | *“Stopped doing things such as clapping and waving”* |
| Lack of social imitation | N/A |  | *“Infrequent imitation of… smiles, facial expression[s]”* |
| Other | N/A |  | N/A |
| **Stereotyped/restricted behaviour** |  |  |  |
| Stereotyped movements | *“Used to lie on his back staring at the ceiling circling his hands and flexing his feet”* |  | *“Started doing several repetitive actions such as moving/twisting his hand in front of his face, flicking the legs of a particular toy over and over or flipping the corner of the floor mat over”* |
| Need for routine/rituals | *“He loved routine, you could not put him in his brother's cot, had to be his own”* |  | *“She would love to line up the DVD cases along the floor”* |
| Stereotyped/restricted interests | *“Didn't watch TV unless credits were rolling then fixated”* |  | *“Particular interest in shoes/Velcro”* |
| Preoccupation with parts of objects | N/A |  | *“Repetitive play behaviour - looking at wheels of toys”* |
| Hypo/hypersensitivity | *“Liked to be wrapped very tightly”* |  | *“She would be sensitive and cry to sudden noises”* |
| **Motor development** |  |  |  |
| Motor delay | *“Couldn't hold head up until 4 months old”* |  | *“Was late to sit, crawl, trying to stand”* |
| Hypotonia | *“Very floppy”* |  | *“He had hypermobile joints”* |
| Hypertonia | *“Pronounced arching of his back”* |  | *“Was very strong, like[d] to stand all the time on tiptoes”* |
| Motor regression | N/A |  | *“At ~8 months stopped weight bearing - would curl legs up, not stand”* |
| Lack of motor imitation | N/A |  | *“Stopped copying”* |
| Swallowing/Sucking | *“Could not swallow his milk”* |  | *“Could not swallow solids”* |
| Other | N/A |  | N/A |
| **Behaviour/Temperament** |  |  |  |
| Lack of attention and interest | *“He will sit and just stay there, staring into nothing”* |  | *“Didn't play with toys”* |
| Hyperactivity | *“Very wiggly active and restless”* |  | *“[Child] always wanted to be on the move”* |
| Passivity | *“Passive in nature”* |  | *“Quiet baby who did not overly seek attention.”* |
| Tantrums/opposition | *“Screamed after feeding”* |  | *“She did become overly angry easy”* |
| Unsettled/crying/anxiety | *“Fussy and hard to comfort at times”* |  | *“Still cried a lot”* |
| Aggression/violence | N/A |  | *“Rough with other children”* |
| Self-harm | *“Would knock head against wall from about 5 months of age”* |  | *“He… tend[ed] to hurt himself on purpose”* |
| Extreme attachment to caregiver | *“Only wanted to be held by Mum, Dad, Grandma, and [Grandma’s husband]”* |  | *“Didn't like being left and leaving main carer”* |
| Other | N/A |  | N/A |
| **Medical issues** |  |  |  |
| Disorder | *“At 5 months she had seizures and they discovered she had several medical issues and developmental issues”* |  | *“West syndrome - seizures become more severe”* |
| Sickness | *“She had bad reflux and would often [vomit]”* |  | *“had a lot of reoccurring ear infections”* |
| Other | N/A |  | N/A |
| **Abnormal physiological function** |  |  |  |
| Sleeping | *“Sleep pattern was terrible”* |  | *“Would wake frequently throughout night (4-5 times) at 12 months”* |
| Feeding | *“Could not keep milk down.”* |  | *“Still not interested in food”* |
| **Atypical physical features** | *“He had a very large head circumference”* |  | *“Bigger and older appearance than his age”* |
| **Unspecified concerns related to autism** | *“Could tell he had lots of similarities to his brother who had been diagnosed with autism and GDD”* |  | *“Her mum was suspicious that she has autism”* |
| **Other idiosyncratic development** | *“She was slow in her milestones”* |  | *“Delayed in all milestones.”* |

Note: Items in bold are domains while indented, non-bolded items are subdomains. There were no subdomains for atypical physical features, unspecified concerns related to autism, and other idiosyncratic development. Quotes are not provided for the “other” subdomains or any subdomains in which parents did not report atypical development at that particular age.

Table S2.

*Negative binomial regressions for the effect of child sex, parent education, and family income on the number of parent-reported areas of atypical development at ≤ 6 months and 7-12 months.*

| Demographic characteristic | Incidence rate ratio | 95% confidence intervals | *p* |
| --- | --- | --- | --- |
| Child sex ≤ 6 months  Female:Male | 0.99 | 0.69 – 0.41 | 0.95 |
| Child sex 7-12 months  Female:Male | 1:00 | 0.76 – 1.31 | 0.99 |
| Older sibling with autism ≤ 6 months |  |  |  |
| Older sibling: no older sibling | 0.74 | 0.47 – 1.15 | 0.18 |
| Older sibling with autism ≤ 6 months |  |  |  |
| Older sibling: no older sibling | 0.95 | 0.69 – 1.30 | 0.75 |
| Parent education ≤ 6 months  Diploma/trade: ≤ 12 years  University:≤ 12 years | 0.84 0.93 | 0.55 – 1.29 0.65 – 1.34 | 0.43  0.71 |
| Parent education7-12 months  Diploma/trade:≤ 12 years  University:≤ 12 years | 1.07  0.89 | 0.78 – 1.46 0.67 – 1.17 | 0.69  0.40 |
| Family income≤ 6 months  $60,001-$104,000:≤ $60,000  >$104,000:≤ $60,000  Prefer not to say:≤ $60,000 | 0.71  0.94  0.76 | 0.47 – 1.08  0.64 – 1.38  0.44 – 1.31 | 0.12  0.75  0.32 |
| Family income7-12 months  $60,001-$104,000:≤ $60,000  >$104,000:≤ $60,000  Prefer not to say:≤ $60,000 | 0.97  0.99  0.66 | 0.71 – 1.32  0.74 – 1.32  0.42 – 1.01 | 0.84  0.92  0.06 |
